# An intrusion detection algorithm for sensor network based on normalized cut spectral clustering

**DOI:** 10.1371/journal.pone.0221920

**Published:** 2019-10-04

**Authors:** Gaoming Yang, Xu Yu, Lingwei Xu, Yu Xin, Xianjin Fang

**Affiliations:** 1 School of Computer Science & Engineering, Anhui University of Science & Technology, Huainan, Anhui, China; 2 Department of Information Science & Technology, Qingdao University of Science & Technology, Qingdao, Shandong, China; 3 Faculty of Electrical Engineering and Computer Science, Ningbo University, Ningbo, China; RMIT University, AUSTRALIA

## Abstract

Sensor network intrusion detection has attracted extensive attention. However, previous intrusion detection methods face the highly imbalanced attack class distribution problem, and they may not achieve a satisfactory performance. To solve this problem, we propose a new intrusion detection algorithm based on normalized cut spectral clustering for sensor network in this paper. The main aim is to reduce the imbalance degree among classes in an intrusion detection system. First, we design a normalized cut spectral clustering to reduce the imbalance degree between every two classes in the intrusion detection data set. Second, we train a network intrusion detection classifier on the new data set. Finally, we do extensive experiments and analyze the experimental results in detail. Simulation experiments show that our algorithm can reduce the imbalance degree among classes and reserves the distribution of the original data on the one hand, and improve effectively the detection performance on the other hand.

## Introduction

With the development of the wireless sensor network (WSN), human activities have been facilitated greatly in recent years. However, WSN also causes many unsafe factors such as privacy and property safety to human. Therefore, the sensor network security has attracted extensive attention, and it has also become an hot topic in the research field of computer science. Just as its name implies, network intrusion detection systems (IDS) is to detect the intrusion behaviors [1, 2]. By analyzing information collected from the key points of a computer network or a computer system, IDS can find out whether there are behaviors breaching a certain security strategy and signs of attack, and the purpose is to effectively protect the privacy and property safety.

Since the network intrusion detection problem can be boiled down to classifying the network connection behavior, many classical pattern classification algorithms have been applied in the network intrusion detection [3]. Commonly used methods are Decision Trees (DT) [4, 5], Gaussian mixture model(GMM) [6], Random Forest(RF) [7], and Support Vector Machines(SVM) [8, 9] etc. Such as, MIsmail et al. [10] proposed two intrusion detection approaches based on multi-level clustering, which combine spectral clustering (SC) and deep neural network (DNN). Ma et al. [11] proposed a novel intrusion detection approach. A new SVM-KNN-PSO intrusion detection method was proposed in [12]. Based on the multi-class SVM classifier, [13] proposed a Fuzzy Multi-class Support Vector Machine (FMSVM) algorithm by introducing fuzzy membership functions. Yang et al. [14] proposed an intrusion detection algorithm, namely CVSGGDI, which denoted the Classification algorithm by a Virtual Sample Generation method based on Gaussian Distribution on Imbalanced data sets. Tayal et al. [15] proposed a RankRC (Ranksvm on Rare Class) algorithm to solve the problems of imbalanced data classification. With the development of mobile communication, WSN is also facing great challenges [16–18]. Deng et al. [19] investigated mobile network intrusion detection for WSN based on a transfer learning.

The previous intrusion detection methods, however, may not achieve a satisfactory performance as they always face such a problem as highly imbalanced attack class distribution. Thus there is still room for improvement with regard to the performance of the intrusion detection algorithm. This paper proposes a Network Intrusion Detection algorithm based on Normalized Cut Spectral Clustering (NIDNCSC) to solve the imbalanced distribution of network connecting behavior. The main contributions are as follows:

Firstly, we select every two classes in the intrusion detection data set to do classification and the algorithm divides the original network connecting samples of the majority class into a relatively small number of clusters.

Secondly, we compute the mean in each cluster as the new training sample and replace the original samples with the means.

Finally, we use all the new training samples and the minority class samples to train a classifier. The purpose is to reduce the imbalance degree among classes and reserves the original distribution of data set.

The rest of this paper organized as follow. We first introduce the spectral clustering algorithm In Section 2. Then a detailed definition of the proposed NIDNCSC algorithm is given in Section 3. The performance of the proposed algorithm was tested in Section 4. We use the KDD CUP 99 data set to test the proposed algorithm, and give the experimental result and detailed analysis in Section 5. Finally, we conclude the paper in Section 6.

## Related works

Based on spectral graph theory, Wu et al. [20] explored the spectral clustering algorithm. Compared with the traditional clustering algorithms, it can cluster on any shape sample spaces and converge to a globally optimal solution. Therefore, the spectral clustering algorithm is originally used in the field of Computer Vision and Very Large Scale Integration(VLSI) design. Recently, it began to be used in machine learning, and soon became a hot research field internationally.

For spectral clustering algorithm, each sample is regarded as a vertex *V* of a graph in the data set, and every pair of nodes (*i*, *j*) can form an edge. In general, we use *w*(*i*, *j*) to denote the weight of the edge (*i*, *j*), and a weighted undirected graph denoted as *G*(*V*, *E*), and then the spectral clustering problem can be boiled down to dividing a graph.

Obviously, in the grouping, the set of vertices need to be partitioned into disjoint sets *V*_1_, *V*_2_,…, *V*_*m*_. On one hand, the vertices belonging to the same set should possess a high similarity, and on the other hand, the vertices belonging to different sets should possess a low similarity. A good grouping method draws lessons from graph partitioning theory.

If the edges between the two subgraphs are removed, the original graph *G*(*V*, *E*) will be divided into two disjoint sets A and B. Both A and B subject to *A* ⋃ *B* = *V*, *A* ⋂ *B* = Φ. In order to control the effect of the partition, a cut function is defined in the following

$$\begin{array}{c} cut\left(A,B\right)=\sum_{u\in A,v\in B}w\left(u,v\right) \end{array}$$ (1)

where *u* and *v* are two vectors belonging to subgraph *A* and *B*, respectively.

**Fig 1:**
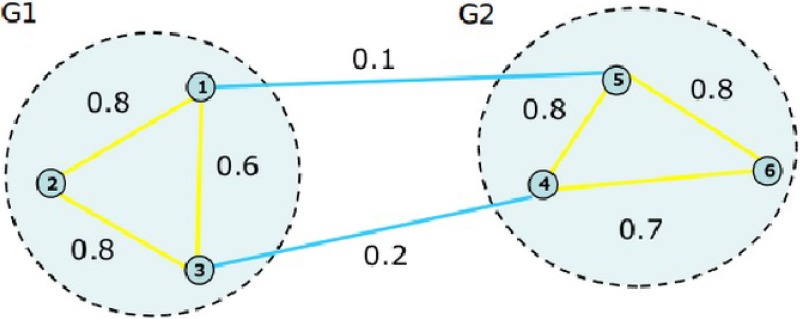
The minimum cut criteria illustration.

The optimal bi-partitioning result corresponds to the minimum cut function value, which is called minimum cut criteria. Fig 1 shows the process. The sample data are represented as nodes and the similarities among samples are denoted by the weights of the undirected edges. We can obtain two clusters by removing the edge between Node ① and Node ⑤, and the edge between Node ③ and Node ④. Obviously, this partition implements the smallest similarity between the two clusters and the largest similarity in each cluster.

However, for the minimum cut criteria, Wu et al. [18] noticed that it favors cutting small sets of isolated nodes in the graph. To overcome this drawback, Shi et al. [21] proposed the normalized cut (Ncut) criteria. The Ncut criteria are as follows.

$$\begin{array}{c} Ncut\left(A,B\right)=\frac{cut(A,B)}{asso(A,V)}+\frac{cut(A,B)}{asso(B,V)} \end{array}$$ (2)

$$\begin{array}{c} asso\left(A,V\right)=\sum_{u\in A,t\in V}w\left(u,t\right) \end{array}$$ (3)

$$\begin{array}{c} asso\left(B,V\right)=\sum_{u\in B,t\in V}w\left(u,t\right) \end{array}$$ (4)

**Fig 2:**
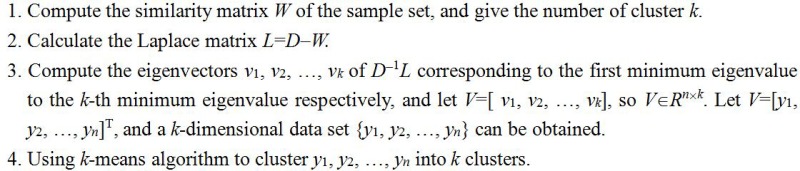
The NCSC algorithm.

Currently, the Normalized Cut Spectral Clustering algorithm (NCSC) is the most popular clustering algorithms and we give the detailed process in Fig 2.

## The NIDNCSC algorithm

**Fig 3:**
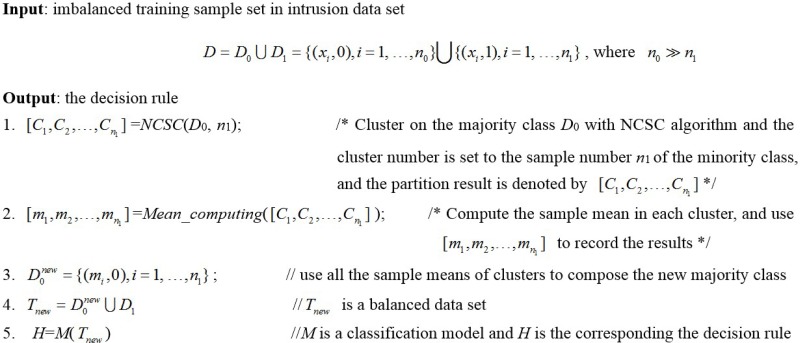
The NIDNCSC algorithm.

In view of the imbalanced data distribution of network connecting behaviors, we propose a Network Intrusion Detection algorithm based on Normalized Cut Spectral Clustering (NIDNCSC) in this section. Firstly, we select every two classes in the intrusion detection data set to do classification, and cluster the samples of the majority class using the normalized cut spectral clustering algorithm. Note that the number of clusters is the same as the number of samples in the minority class. After clustering, we compute the mean of each cluster, which denoted as *m*_1_, *m*_2_,…, *m*_*k*_, and then use all the means to construct the new training set of the majority class. Finally, we use the original minority class samples and the new majority class samples to train a classifier. The pseudo-code of the NIDNCSC algorithm is given in Fig 3.

**Fig 4:**
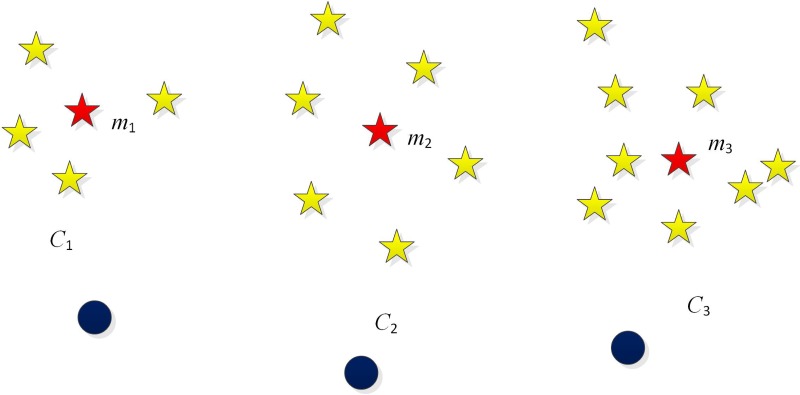
An illustration on the NIDNCSC algorithm.

An illustration on the NIDNCSC algorithm is given in Fig 4, where yellow five-pointed stars and blue circle points are two classes. The yellow five-pointed stars are the majority class, and the blue circle points are the minority class. As shown in Fig 4, the NIDNCSC algorithm cluster the majority class into three clusters, where the number 3 equals the sample number of the minority class. The three clusters are denoted by *C*_1_, *C*_2_, *C*_3_. Since the majority class samples are replaced by the means of all the clusters, i.e., *m*_1_, *m*_2_, and *m*_3_, the size of the new majority class are equal to that of the minority class. Thus, the NIDNCSC algorithm can effectively reduce the imbalance among different classes. Furthermore, since *m*_1_, *m*_2_, and *m*_3_ are the means of the clusters, the distribution information of the majority class can be reserved approximately. Consequently, the proposed NIDNCSC algorithm is expected to win a good intrusion detection performance.

## Experiments on two artificial data sets

To verify whether the NIDNCSC algorithm can deal with imbalanced data classification problem, we use two artificial imbalanced data sets to conduct extensive experiments in this section. In each experiment, we use the SVM classifiers as the basic classifier and they are implemented by LIBSVM software [22].

### The experimental data set introduction

**Fig 5:**
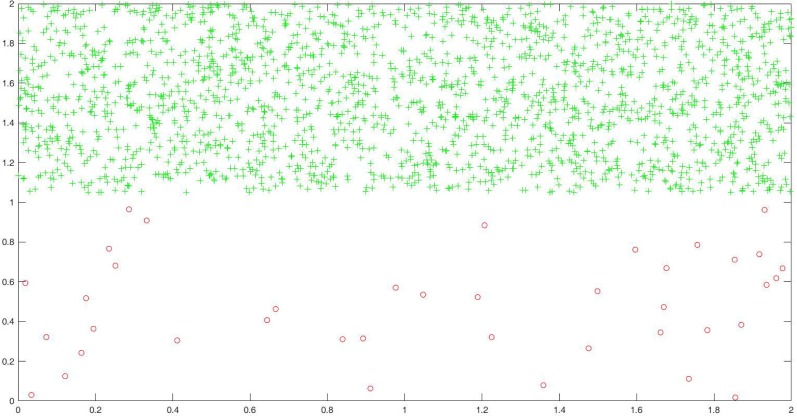
The training sample distribution of the first artificial data set.

In this part, the artificial data sets are constructed by the following approach. For the first artificial data set, we randomly generate two classes samples using two different uniform distributions. We use the distribution of *U*([0, 2] × [0, 0.95]) to generate Class One, and the distribution of *U*([0, 2] × [1.05, 2]) to generate Class Two. For Class One, we randomly generate 60 samples, and randomly select 40 samples as training samples, the rest as testing samples. For Class Two, we generate 3000 samples, and randomly select 2000 samples as training samples, the rest as testing samples. Obviously, the sample number of Class Two is 50 times that of Class One, so it is an imbalanced data set. Fig 5 shows the distribution of training samples, where the green asterisk point denotes the majority class, and the red circle point denotes the minority class.

**Fig 6:**
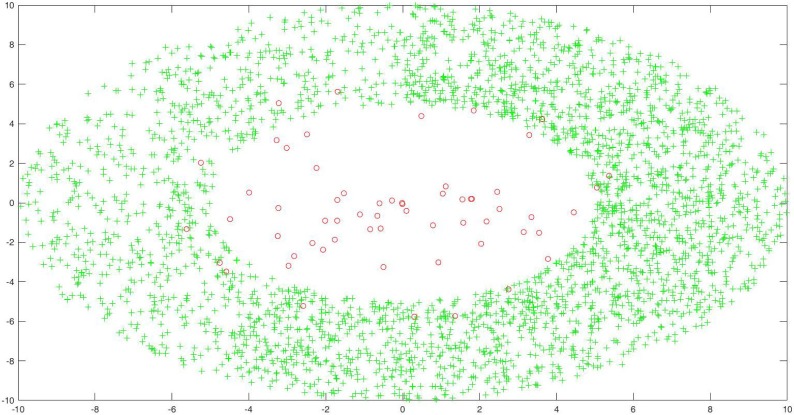
The training sample distribution of the second artificial data set.

For the second artificial data set, we generate two classes of concentric samples as following.

$$\begin{array}{c} \left\{ \begin{array}{cc} & x=\rho \;\text{cos}\;\theta \\ & y=\rho \;\text{sin}\;\theta \end{array}\right. \end{array}$$ (5)

where *θ* ∈ *U*[0, 2*π*] for Class One and Two, *ρ* ∈ *U*[0, 6] for Class One, and *ρ* ∈ [5, 10] for Class Two. For Class One, we generate 100 samples and randomly select 60 samples as training samples, and the rest as testing samples. For Class Two, we randomly generate 5000 samples and randomly select 3000 samples as training samples, the rest as testing samples. Just as the first artificial data set, it is also an imbalanced one. Fig 6 shows the distribution of training samples, where the green asterisk point denotes the majority class, and the red circle point denotes the minority class.

### Review of the SMOTE algorithm

**Fig 7:**
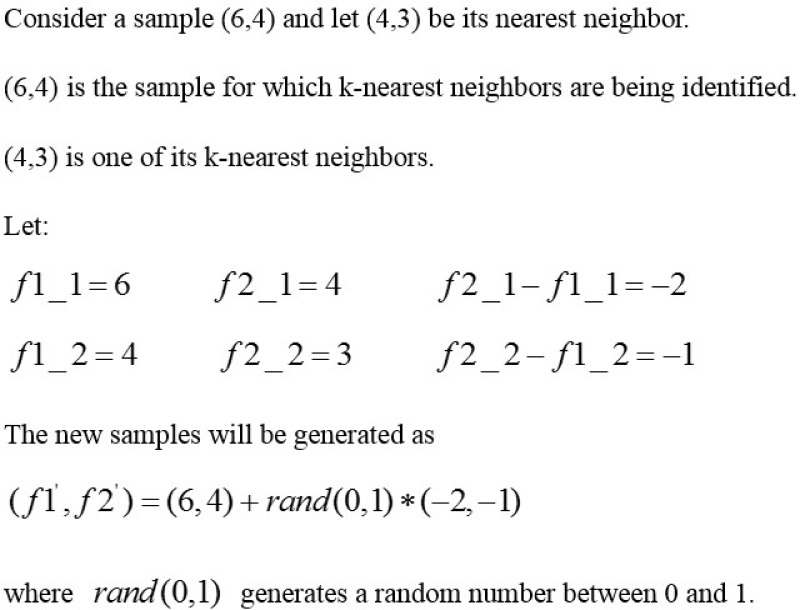
Generation example of synthetic examples.

To verify the validity, we compare our algorithm with the Synthetic Minority Over-sampling Technique (SMOTE) algorithm [23], which creates synthetic samples in feature space rather than data space. The detailed generation process of synthetic samples is as follows. Firstly, for each sample *p* in the minority class, SMOTE algorithm compute the *k* nearest neighbors from the minority class. Then, according to the amount of over-sampling *d*, they choose a certain number (*l* ≤ *k*) of neighbors from the *k* nearest neighbors. Finally, for each sample *q* from the *l* neighbors, SMOTE algorithm choose a sample randomly from the line segment that connects *p* and *q*. For instance, if *k* = 6 and *d* = 300%, then SMOTE algorithm select three samples from the six nearest neighbors, and for each sample, SMOTE algorithm can generate a synthetic sample by choosing a point from the corresponding line segment. Fig 7 shows a calculation example of random synthetic samples.

### Experimental methods and evaluation metric

**Fig 8:**
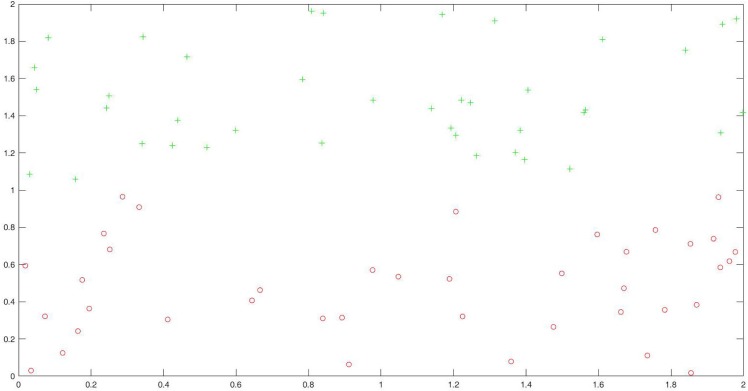
The new data distribution acquired by the NIDNCSC algorithm for the first artificial data set.

**Fig 9:**
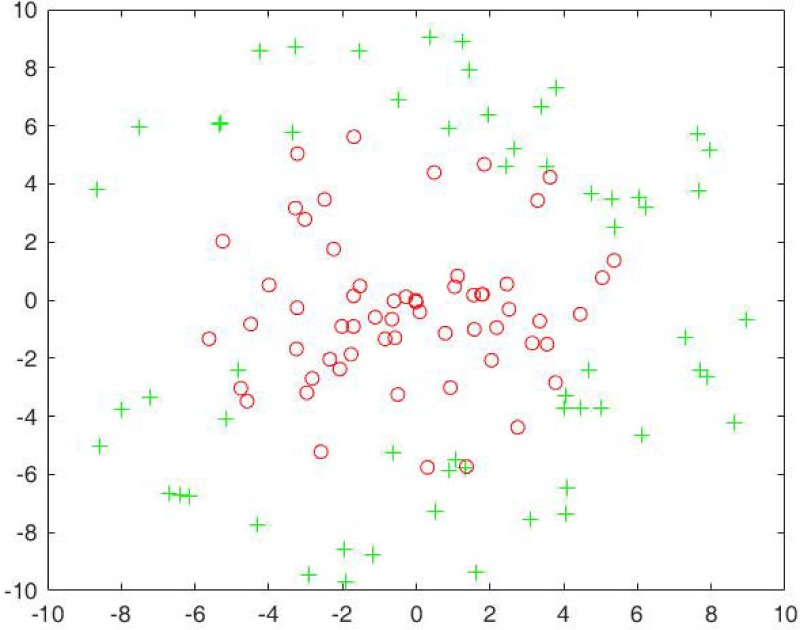
The new data distribution acquired by the NIDNCSC algorithm for the second artificial data set.

We compare our algorithm with SMOTE algorithm and RankRC algorithm. Since the RankRC algorithm does not preprocess the original data set, we only use the SMOTE algorithm and the NIDNCSC algorithm to do data preprocessing. For the SMOTE algorithm, we set *k* = 60, and the amount of over-sampling needed is set to 4900%. Thus, we choose 49 neighbors from the 60 nearest neighbors randomly and generate 49 synthetic samples for each sample in the minority class. As a result, the size of the new minority set obtained by the SMOTE algorithm is equal to the size of the original majority class. For the proposed NIDNCSC algorithm, we first cluster the majority class into 60 clusters and then replace the original samples in each cluster with the mean sample. Therefore, the obtained data set is also a balanced one. Figs 8 and 9 show the distribution of the new data set acquired by the NIDNCSC algorithm for the two artificial data sets, respectively.

We select the SVM model for classification, which computes the following optimization problem for the best classification hyperplane.

$$\begin{array}{c} \begin{array}{cc} \min_{w,b,\xi } & \;\frac{1}{2}\|w{\|}^{2}+C\sum_{i=1}^{l}\xi_{i}\\ s.t. & \;y_{i}\left(\left(w\cdot \Phi \left(x_{i}\right)\right)+b\right)\geq 1-\xi_{i},\;i=1,\ldots l\\ & \xi_{i}\geq 0,\;i=1,\ldots l \end{array} \end{array}$$ (6)

where *ξ*_*i*_ ≥ 0, *i* = 1,,…*l* are the slack variables, *C* > 0 is trade-off factor between the margin and the slack variable penalty, Φ is a mapping function from the original sample space to the high dimensional feature space, *w* is the normal vector, and *b* is the bias of the hyperplane. We choose the following radial basis function (RBF)

$$\begin{array}{c} K\left(x,y\right)=\exp\left(\frac{-\|x-y{\|}^{2}}{2\sigma^{2}}\right) \end{array}$$ (7)

as the kernel function, where *σ* is a width parameter. The most suitable value for the width parameter *σ* and the penalty factor *C* in the SVM model is calculated by 10-folds cross-validation. Precision and recall are chosen as the metrics to evaluate the three different algorithms. Obviously, if both precision and recall rates are higher, the algorithm achieves a better performance.

### Experimental results and analysis

We perform five runs of 10-fold cross-validation for each algorithm. Tables 1 and 2 shows the average result.

**Table 1:** Classification performance on the first artificial data set.

Classification algorithm	Precision(%)	Recall(%)	Running time (s)	*σ*	C
SMOTE	93.6	90.6	12.2	128	100
RankRC	93.2	90.1	8.6	64	50
NIDNCSC	95.2	92.2	5.3	16	200

**Table 2:** Classification performance on the second artificial data set.

Classification algorithm	Precision(%)	Recall(%)	Running time (s)	*σ*	C
SMOTE	90.1	88.2	20.3	256	1000
RankRC	89.9	88.0	12.8	32	200
NIDNCSC	93.3	90.6	8.2	32	500

From Tables 1 and 2, we can know that the NIDNCSC algorithm can achieve the highest precision and recall rates and moreover it costs the least running time. This is because the NIDNCSC algorithm can reduce the imbalance of the data set and reserve the data distribution of the majority class. Though the SMOTE algorithm can also reduce the imbalance by generating artificial samples, it cannot reserve the data distribution as the generated artificial samples destroy the original distribution. Though the RankRC algorithm optimize AUC in the training process, the data set used for training is also imbalanced, which leads to the precision and recall rates the lowest. Further, the proposed NIDNCSC algorithm is the most efficient among these algorithms. This is because that NIDNCSC algorithm reduces the size of the training set while the SMOTE algorithm enlarges that. In addition, the RankRC algorithm employs a minority class kernel representation to train a classifier on the original data set; it can also obtain relatively high efficiency.

## The network intrusion detection experiment

In this section, we use the KDDCup_10_per data set [22] to test the performance of the NIDNCSC algorithm. This data set consists of 494021 TCP connections and every TCP connection contains 41 features. 34 features are continuous, and the rest 7 ones are discrete. It includes 23 types, where the label ‘normal’ denotes the normal network link records and the other 22 types are all network intrusion records, such as ‘Neptune’, ‘Back’, or ‘Smurf’. We first map all the 23 types into 5 large classes, that is, ‘Normal’, ‘R2L’, ‘U2R’, ‘Dos’, and ‘Probing’. The data distributions of the five large classes are listed in Table 3. The experimental environment used in this experiment is identical with that of the above experiment.

**Table 3:** Data distributions of different classes.

Class	Sample number	Percentage(%)
Normal	97278	19.69
Dos	391458	79.24
Probing	4107	90.6
R2L	1126	0.23
U2R	52	0.01

### The experimental data construction

In this experiment, we compare the NIDNCSC algorithm with the FMSVM algorithm, the CVSGGDI algorithm, and the RankRC algorithm. As the KDDCup_10_per data set is too large, for simplicity, we just select a small proportion of samples from each class to construct a data set I for the experiment. The detailed information is shown in Table 4. Clearly, the data set I is an imbalanced data set.

**Table 4:** The distribution of data set I.

Class	Training sample number	Testing sample number(%)
Normal	900	960
Dos	3700	3790
Probing	600	800
R2L	300	398
U2R	30	22

We use data set I to compare the FMSVM algorithm and the RankRC algorithm. With respect to the CVSGGDI algorithm, we generate a new data set II based on data set I [14]. The detailed information on the new data set II is shown in Table 5. We use it to test the CVSGGDI algorithm.

**Table 5:** The distribution of data set II.

Class	Training sample number	Testing sample number(%)
Normal	900	960
Dos	3700	3790
Probing	600	800
R2L	1200	398
U2R	330	22

For the NIDNCSC algorithm, we first use every two classes in the intrusion detection to do classification, and then set the cluster number in the majority class equal to the size of the minority class. For example, if training a classifier between Probing and U2L, we can set the cluster number equal to 20, and then use the NIDNCSC algorithm to cluster the majority class, namely Probing and compute the mean value of each cluster. Finally, we use the mean value of each cluster to replace the original samples. Meanwhile, we keep the minority class unchanged, label the constructed data set as III and use it to test the proposed NIDNCSC algorithm.

### Data preprocessing

In order to reduce the influence of the attribute value measure of KDDCup_10_per data set on the experimental results, we need to normalize the input of the support vector machine. Therefore, we use min-max normalization to convert the character attribute values of the KDDCup_10_per data set into numerical attribute values. The normalized formula is as follows.

$$\begin{array}{c} V=\frac{v-\min(f_{i})}{\max\left(f_{i}\right)-\min\left(f_{i}\right)} \end{array}$$ (8)

where *v* is the original data set attribute value, *V* is the normalized attribute value, and max(*f*_*i*_), min(*f*_*i*_) are the maximum values and minimum values of attribute *f*_*i*_, respectively.

### The performances metric and experimental result

In order to better compare the performance of different intrusion detection algorithms, we use the detection rate and false alarm rate as performance metrics. The computation formulas of detection rate (DR) and the false alarm rate (FR) are as follows.

$$\begin{array}{c} DR=\frac{d_{1}}{D_{1}},\;FR=\frac{d_{2}}{D_{2}} \end{array}$$ (9)

where *d*_1_ and *d*_2_ are the number of abnormal samples detected and the number of misclassification normal samples, respectively. *D*_1_ and *D*_2_ are the total number of abnormal samples and the total number of normal samples, respectively.

**Fig 10:**
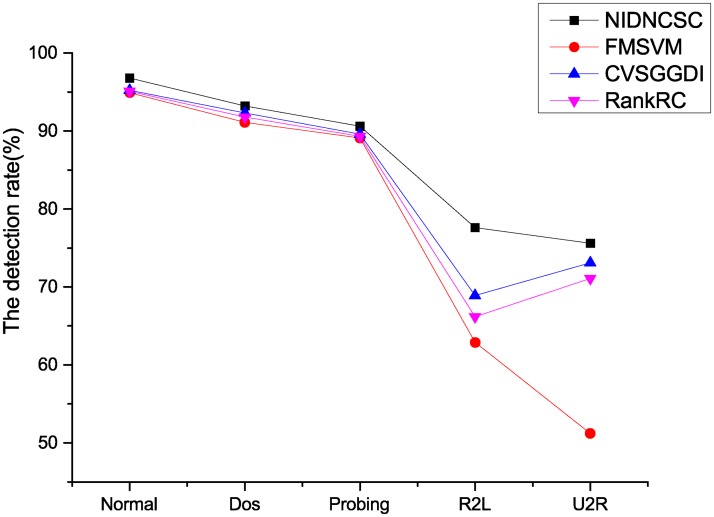
Comparison of the precision rate.

**Fig 11:**
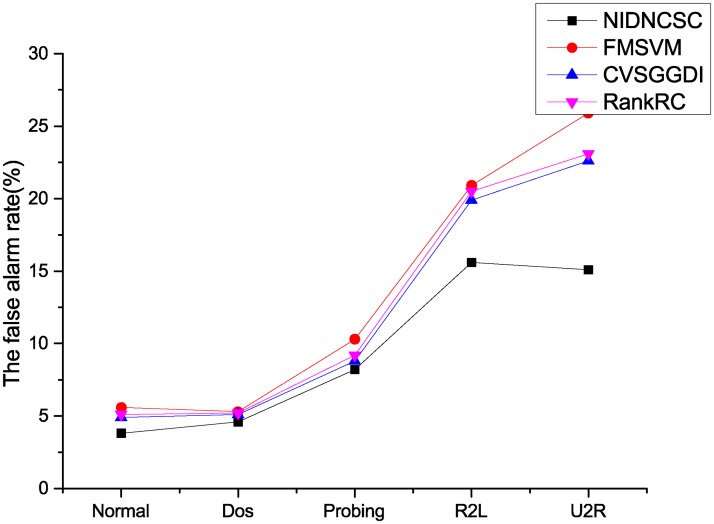
Comparison of the false alarm rate.

Here, we compare the NIDNCSC algorithm with the FMSVM algorithm, the CVSGGDI algorithm, and the RankRC algorithm. Because the network intrusion detection data set contains five classes, we can think of it as a multi-class problem, and use the one against all (1-v-r) method to deal with the problem [24]. Figs 10 and 11 and Table 6 show the experimental results.

**Table 6:** The best experimental parameter values and running time comparison.

Algorithms	Running time (s)	*σ*	C
NIDNCSC	56.2	256	200
FMSVM	70.1	32	50
CVSGGDI	82.3	1024	100
RankRC	66.2	128	50

From Figs 10 and 11 and Table 6, we can know that the NIDNCSC algorithm A has the best detection performance compared to the FMSVM, the CVSGGDI algorithm and the RankRC algorithm. In detail, though the performance improvements on ‘Normal’, ‘Dos’, and ‘Probing’ are not very clear, the performance improvements on ‘R2L’ and ‘U2R’ are obvious. In addition, the NIDNCSC algorithm wins the best running efficiency. This is because that the NIDNCSC algorithm reduces the imbalance by clustering on the majority class, and reserves the data distribution of the majority class. However, the FMSVM and the RankRC do not consider the imbalance between the classes. Though the CVSGGDI algorithm can also reduce the imbalance, it needs to generate extra virtual samples, so the running efficiency is low.

## Conclusions

In this paper, we propose the NIDNCSC algorithm, which can both reduce the data set imbalance and reserve the data distribution of the majority class, so it can obtain a good detection performance than the previous network intrusion detection algorithm. Experiments also show the effectiveness of the NIDNCSC algorithm.
